# A hydrophobic spine stabilizes a surface-exposed α-helix according to analysis of the solvent-accessible surface area

**DOI:** 10.1186/s12859-016-1368-z

**Published:** 2016-12-22

**Authors:** Yi-Fan Liou, Hui-Ling Huang, Shinn-Ying Ho

**Affiliations:** 1 Institute of Bioinformatics and Systems Biology, National Chiao Tung University, Hsinchu, Taiwan; 2 Department of Biological Science and Technology, National Chiao Tung University, Hsinchu, Taiwan

**Keywords:** Hydrophobic spine, Molecular dynamics simulation, Physicochemical properties, Protein folding, Solvent-accessible surface area, Support vector regression, Knowledge discovery

## Abstract

**Background:**

Most of hydrophilic and hydrophobic residues are thought to be exposed and buried in proteins, respectively. In contrast to the majority of the existing studies on protein folding characteristics using protein structures, in this study, our aim was to design predictors for estimating relative solvent accessibility (RSA) of amino acid residues to discover protein folding characteristics from sequences.

**Methods:**

The proposed 20 real-value RSA predictors were designed on the basis of the support vector regression method with a set of informative physicochemical properties (PCPs) obtained by means of an optimal feature selection algorithm. Then, molecular dynamics simulations were performed for validating the knowledge discovered by analysis of the selected PCPs.

**Results:**

The RSA predictors had the mean absolute error of 14.11% and a correlation coefficient of 0.69, better than the existing predictors. The hydrophilic-residue predictors preferred PCPs of buried amino acid residues to PCPs of exposed ones as prediction features. A hydrophobic spine composed of exposed hydrophobic residues of an α-helix was discovered by analyzing the PCPs of RSA predictors corresponding to hydrophobic residues. For example, the results of a molecular dynamics simulation of wild-type sequences and their mutants showed that proteins 1MOF and 2WRP_H16I (Protein Data Bank IDs), which have a perfectly hydrophobic spine, have more stable structures than 1MOF_I54D and 2WRP do (which do not have a perfectly hydrophobic spine).

**Conclusions:**

We identified informative PCPs to design high-performance RSA predictors and to analyze these PCPs for identification of novel protein folding characteristics. A hydrophobic spine in a protein can help to stabilize exposed α-helices.

**Electronic supplementary material:**

The online version of this article (doi:10.1186/s12859-016-1368-z) contains supplementary material, which is available to authorized users.

## Background

Prediction of the dominant fold of proteins and discovery of protein folding characteristics in an aqueous solution have been challenging problems recently [1, 2] although many methods, such as molecular dynamics simulations, folding recognition, and homology modeling, have been used to study protein folding in recent years. To elucidate folding states of proteins, estimation of accessible surface areas (ASAs) is a simple method to determine whether a residue is buried or exposed and hence the function of this residue can be ascertained. Therefore, the ASA is considered a crucial factor for prediction of protein structure. Predicting the ASA is an important approach in studies on the structure and function of proteins.

Hikijata *et al.* [3] predicted three-dimensional (3D) structures of proteins using alignment results and solvent accessibility of residues. Huang *et al.* [4] indicated that the ASA is useful for identification of DNA-binding domains in sequences. Zhu and Blundell [5] analyzed secondary structures of proteins and found amino acid patterns of solvent-inaccessible faces of α-helices and solvent-accessible sides of β-strands. Kumar and Bansal [6] analyzed α-helices in globular proteins and suggested that Ncap is mostly composed of Ser, Asp, Thr, Asn, Gly, and Pro. Pascarella *et al*. [7] and Bartlett *et al*. [8] used 3D structure information to study solvent accessibility of residues and characteristics of catalytic sites, respectively. Shirota *et al*. [9] estimated the surface-to-volume ratio of residues to examine the sequence-structure relation.

Barton502 is a dataset that is used lately to study and predict secondary structures. Barton502 contains protein structures that were chosen using strict conditions [10] and then was used to estimate relative solvent accessibility (RSA) of proteins in many studies. Table 1 shows some relevant studies on real-value RSA prediction using various regression methods, such as support vector regression (SVR), multilayer regression (MLR), neural network (NN), and information theory [11–20]. Among these machine learning methods, the NN is the first method to be tested for predicting protein solvent accessibility and is still extensively employed in various studies. SVR is another effective method for the RSA prediction. Several features were selected to train these machine learning models, such as local residue composition, probability profiles, and a position-specific scoring matrix (PSSM). Although RSAs of residues are known to be closely linked with protein functions, few researchers are studying protein folding characteristics using sequence-based RSA predictors.

**Table 1:** Relevant studies on real-value RSA prediction

Reference	Year	Regression method	Features
Ahmad [11]	2003	NN	Amino acid proportions
Yuan [12]	2004	SVR	Amino acid proportions
Adamczak [13]	2004	NN	PSSM
Wang [14]	2005	MLR	Amino acid proportions, PSSM, and sequence length
Garg [15]	2005	NN	PSSM and secondary structure
Nguyen [16]	2006	Two-stage SVR	PSSM
Chang [17]	2008	Two-stage SVR	enhance PSSM and sequence length
Iqbal [18]	2015	Basic exact regression	PSSM, PCPs and disorder probability
Fan [19]	2015	GBRT	PSSM, secondary structure, and native disorder
Zhang [20]	2015	SVR	PSSM, PCPs, secondary structure, disorder probability
SVR-RSA	2016	SVR	PSSM, PCPs, and sequence length

Tung and Ho [21] proposed an informative property-mining algorithm that involves an inheritable biobjective combinatorial genetic algorithm (IBCGA) [21] to select informative physicochemical properties (PCPs) to predict immunogenicity of MHC class I-binding peptides. In the present study, our aim was to design a high-performance predictor of RSA using SVR with informative PCPs obtained by means of IBCGA with SVR to identify new protein folding characteristics. These features were combined with informative PCPs, PSSMs, and sequence length, and the resulting predictor, named SVR-RSA, turned out to be more accurate on the Barton502 dataset than the exiting RSA predictors. The analysis of informative PCPs of residues yielded a special set of exposed hydrophobic residues of an α-helix, named a *hydrophobic spine*. The latter consists of periodically repeating exposed hydrophobic residues: every three or four positions.

To characterize the hydrophobic spine, proteins 1MOF and 2WRP (Protein Data Bank IDs), which have a perfectly hydrophobic spine and an imperfectly hydrophobic spine, respectively, were used as examples to analyze structural stability by molecular dynamics simulations of 10 ns at 300, 400, and 500 K [22]. Two mutants, 1MOF_I54D and 2WRP_H16I, which have an imperfectly hydrophobic spine and a perfectly hydrophobic spine, respectively, were also compared with their wild-type versions. The simulation results revealed that a hydrophobic spine in a protein can help to stabilize exposed α-helices, and this result may be helpful in protein engineering.

## Methods

We used the IBCGA algorithm to select small feature sets of informative PCPs and to discover knowledge by analyzing these feature sets. Each model for one of 20 amino acid residues has its own feature set. The analysis of the informative PCPs deduced the hydrophobic spine, which was further studied using molecular dynamics simulations.

### The dataset

The Barton502 dataset was used for designing the high-performance RSA predictor. Barton502 contains 502 nonhomologous sequences collected by Cuff and Barton [23]. Barton502 was randomly subdivided into a training set and test set, which contain 336 and 166 sequences, respectively. According to Chang *et al*. [17], there are 84 sequences in the training dataset that was randomly selected, named the Sma dataset, for feature selection. Every protein was divided into a number of small segments using a sliding window 11 amino acid residues long [17], where the central residue of the segment is the prediction target while the five nearest bilateral residues provide additional information. All the segments were grouped according to their central residues, and 20 RSA prediction models were built. The real solvent-accessible surface areas were calculated using the DSSP software [24]. According to the definition of Singh and Ahmad [25], the RSA value of a residue was computed by dividing the real ASA value by the value observed in the extended Ala-X-Ala conformation of the residue. In the present study, the ASA value is the main parameter for evaluating the real-value RSA predictors.

### Feature extraction

#### PCPs

The 544 amino acid indices for describing the PCPs were directly downloaded from the AAindex database [26]. The indices containing “N/A” (not available) elements were excluded, and there were 531 indices left. Each averaged value of a property for the 11-meric segment served as a feature value calculated as in a previous study [21]. Therefore, every segment had 531 features of PCPs. The feature values *x* were normalized to [0,1] using the standard logistic function:

1 $$ x\mathit{\hbox{'}} = \frac{1}{1+ \exp \left(-x\right)} $$

#### PSSMs

PSSMs of the sequences in the Barton502 dataset were calculated using the PSI-BLAST software [27]. The settings of PSI-BLAST were as follows: the E-value threshold was 10^−3^, the multipass inclusion E-value threshold was 2 × 10^−3^, and the iteration number was 4. Each residue of a segment was represented by a 21-dimensional vector that contains 20 values representing effective frequencies of occurrence at respective positions in a multiple alignment and an extra value for the terminal flag as described in the article by Chang *et al*. [17]. Finally, the PSSM of a segment was represented by 231 values. The score values were normalized using equation (1).

### IBCGA-SVR

The IBCGA consists of an intelligent genetic algorithm [28] with an inheritable mechanism. The intelligent genetic algorithm can select *r* informative features from a large number *n* of candidate features with the search space of C(*n*,*r*) while optimizing an objection function [21]. Tung and Ho [21] proposed an informative property-mining algorithm that combines IBCGA and support vector classification to identify a small set of informative PCPs and to predict immunogenicity of MHC class I-binding peptides. In the present study, we propose a novel method (named IBCGA-SVR) for selection of informative PCPs on the basis of a combination of IBCGA and ε-SVR by minimizing the mean absolute error (MAE):

2 $$ MAE=\frac{1}{n}{\displaystyle {\sum}_{i=1}^n{V}_i-{V}_i\mathit{\hbox{'}}} $$

where *n* is the number of the predicted segments, and *V* and *V’* are the real and predicted RSA values, respectively. The ε-SVR was obtained from LIBSVM (version 2.84) [29]; the RBF kernel was used.

The population size, cross-over rate, and mutation rate of IBCGA were set to 50, 0.8, and 0.05, respectively [30]. The r_start_ and r_end_ were set to 40 and 10, respectively. The encoded chromosomes were designed as described elsewhere [21]. The gene number was 531, plus three 4-bit genes for tuning parameters *C*, γ, and ε for ε-SVR. The fitness function involved MAE (detailed in the next section). To select robust feature sets, 30 independent runs were performed for each amino acid, and the feature sets having minimal MAE were selected for constructing RSA predictors.

### SVR-RSA

The proposed method SVR-RSA is designed not only to predict RSAs of amino acid residues, but also to select informative PCPs for identification of characteristics of proteins. The design of SVR-RSA includes two steps: selecting informative PCPs using IBCGA-SVR and implementing the RSA predictors based on the informative PCPs.

After selection of the PCP feature sets using IBCGA-SVR, these feature sets were combined with PSSMs and sequence lengths to construct predictors. The corresponding *C*, γ, and ε of the models were optimized using grid search software available in the LIBSVM package. The target residues were predicted utilizing the corresponding model of the 20 different models. To avoid the overfitting problems and for performance comparing, Barton502 is divided into the training and test parts which are respectively utilized for creating the predicting models and evaluating the predicting power for comparisons between the predictors in this study and other RSA predictors built using Barton502.

MAE of the 10-fold cross-validation (10-CV) was calculated both in the IBCGA-SVR and grid search. Pearson’s correlation coefficient was also used for estimating the performance:

3 $$ CC=\frac{1}{n-1}{\displaystyle {\sum}_{i=1}^n\left(\frac{X-\overline{X}}{S_x}\right)\left(\frac{Y-\overline{Y}}{S_y}\right)} $$

where *n* is the total number of residues and *X*, *Y*, $\overline{X}$, and $\overline{Y}$, are the predicted, observed RSA values, the average of the predicted RSA values, and the average of the observed RSA values, respectively.

### Definitions of hydrophobic residues and the α-helix exposure degree

The 20 residues were classified into “hydrophilic” and “hydrophobic” categories using the Kyte-Doolittle index [31] with the threshold of 0. Seven residues, Ala, Cys, Ile, Leu, Met, Phe and Val, were defined as hydrophobic residues, while the other 13, Asp, Glu, Gly, His, Lys, Asn, Pro, Gln, Arg, Ser, Thr, Trp, and Tyr as hydrophilic residues. The residues were also assigned an exposed or buried status according to the RSA values at the threshold of 25% [32]. Hence, all residues of proteins can be defined as exposed hydrophilic, exposed hydrophobic, buried hydrophilic, and buried hydrophobic residues.

The protein secondary structures of Barton502 were all defined using the DSSP software [24]. The α-helix exposure degree was defined as follows:

4 $$ AED=\frac{n_e}{n} $$

where *n*_*e*_ and *n* denote the number of exposed residues and the total number of residues in an α-helix, respectively.

### Thermal stability analysis

Molecular dynamics simulations were carried out in GROMACS v4.5.5 [33]. The OPLS force field [34] was applied in this study. 2WRP and 1MOF were retrieved from the Protein Data Bank (PDB) and the mutant structures, 2WRP_H15I and 1MOF_I54D, were constructed using the PS^2^ web server [35]. The templates were set up using the wild-type 2WRP and 1MOF, respectively, and other parameters were set to default values. These four proteins were supplemented with missing hydrogen atoms and were protonated by considering the protonation state corresponding to pH 7. The periodic boundary conditions were obtained using the dodecahedron box and the minimum distances of a nonhydrogen protein atom to the box wall of at least 1 nm. By means of the single point charge model, water molecules were soaked into the box around the proteins and the counterions were added to neutralize the net charge of the whole simulation system. To ensure that the solvent distribution was kept at the minimum energy, the atoms of proteins were first fixed and subjected to 50,000 iterations of the steepest descent energy minimization, and then 200-ps molecular dynamics [22] simulations were performed for solvation. Next, the protein and solvent molecules were unconstrained and optimized using the steepest descent energy minimization for 50,000 iterations followed by 10-ns molecular dynamics simulations. In the simulation system, the particle-mesh Ewald (PME) method was used for calculating the long-range electrostatics, and all bonds were constrained using the LINCS algorithm. Time steps were set to 2 fs for 5,000,000 iterations for 10-ns simulations. Three simulation temperatures, 300, 400, and 500 K, were applied to the constant temperature and pressure (NPT)-simulated environments using a weak coupling algorithm that had the pressure of 1 atm and pressure coupling time of 1 ps. The temperature coupling was also applied, with 0.1 ps as coupling time. The trajectories were recorded every 2 ps for the analysis.

## Results and discussion

Several real-value RSA predictors using Barton502 were compared in terms of performance. The results showed that the performance of the predictors proposed in this study is better than that of the predictors using Barton502. The informative PCPs were also analyzed. These PCPs indicated that a hydrophobic spine can help to stabilize a protein structure. Two proteins that have a perfectly and imperfectly hydrophobic spine, respectively, and their mutants that show the reverse situation (imperfectly hydrophobic spine and perfectly hydrophobic spine, respectively) were used to conduct several molecular dynamics simulations to validate the hydrophobic spine.

### Performance comparisons among the real-value RSA predictors

The Sma dataset was used for selection of informative PCPs. Hence, the 20 amino acid residues had their PCP feature sets from which we created their own predictors (for each residue). The sequences were first split into small fragments with the length of 11 amino acid residues each. Each fragment was predicted using the corresponding RSA predictors according to the central residue. The PCP numbers selected by means of IBCGA-SVR are listed in Table 2. The range of PCP numbers was from 10 to 31. Among the 20 amino acid residues, the predictive model for Cys appears to have the best MAE (8.59), while the predictive model for Gly has the lowest MAE: 25.19. These results are similar to the those reported elsewhere [19] where the researchers used gradient-boosted regression trees to build the predictive models. Except for Ala, the hydrophobic residues including Cys, Ile, Leu, Met, Phe, and Val have better predictive models, with MAE from 8.59 to 12.83, than most of hydrophilic residues do. These results are in good agreement with the findings of other researchers [19] who supposed that Gly often constitutes the flexible regions of proteins and that other hydrophilic residues play similar roles.

**Table 2:** The PCP feature number of the predictor of RSA for each amino acid residue and MAE of each predictor

Residue	Feature number	MAE (%)^a^	Residue	Feature number	MAE (%)^a^
A	30	18.93	L	22	11.74
R	23	18.87	K	32	16.82
N	31	23.28	M	31	12.24
D	21	22.71	F	29	11.95
C	19	8.59	P	29	19.99
Q	32	19.51	S	32	23.17
E	29	20.90	T	12	21.25
G	25	25.19	W	10	12.17
H	14	18.82	Y	30	14.20
I	30	10.86	V	31	12.83

^a^The MAE for 10-CV of the Sma dataset

These PCPs were then combined with PSSMs and sequence length to build the final RSA-predicting models, and the test results are provided in Table 3. In other studies [11–20], several predictive methods were used, including the NN, multilayer regression, SVR, and two-stage SVR. Those predictors involve six features including PSSM, AAindex, sequence length, amino acid proportions, disorder probability, and secondary structure information. Among the RSA predictors, AAindex was first tested in our study. The real-value RSA predictors based on Barton502 as the dataset are also listed for comparison in Table 3. In this study, the test MAE and correlation coefficient of 14.11 and 0.69, respectively, are slightly better than those of the other RSA predictors (which are based on Barton502).

**Table 3:** The feature usage and a performance summary from other studies that used Barton502 as a dataset

features	Ours	Chang, *et al*. (2008)^a^	Nguyen, *et al*. (2006)^a^	Garg, *et al*. (2005)^a^	Wang, *et al*. (2005)^a^	Yuan, *et al*. (2004)^a^	Ahmad, *et al*. (2003)^a^
PSSM	Yes	Yes	Yes	Yes	Yes	No	No
AAindex (PCPs)	Yes	No	No	No	No	No	No
sequence length	Yes	Yes	No	No	Yes	No	No
amino acid composition	No	No	No	No	No	Yes	Yes
secondary structure	No	No	No	Yes	No	No	No
regression tool	one-stage SVR	two-stage SVR	two-stage SVR	NN	MLR	one-stage SVR	NN
MAE (%)	14.11	14.80	15.70	15.90	16.20	18.50	18.80
CC	0.69	0.68	0.66	0.65	0.64	0.52	0.48

^a^MAE and CC are from the original paper

To compare the predictive models of amino acid residues, the test dataset was also processed using several RSA predictors, including SPINE X [36], SABLE [37], RVP-net [38], and SARpred [15]. The test results are presented in Table 4. RSA predictors for Ala, Asp, Asn, Glu, Gln, Gly, Ile, Leu, Ser, and Tyr showed better performance than the predictors corresponding to the other amino acid residues. The Pearson correlation coefficient of the overall test dataset in this study is also comparable to that of SPINE X, but MAE is slightly better than MAE of SPINE X.

**Table 4:** Performance comparison among real-value RSA predictors

amino acid	ours	Chang [17]	SPINE X	SABLE	RVP-net	SARpred
A	12.22*	13.30	12.52	46.98	18.93	16.10
R	16.81	17.00	**16.15**	26.61	20.31	18.98
N	**18.50**	19.60	18.63	32.00	24.70	22.05
D	**18.08**	19.20	18.21	28.97	23.81	21.99
C	8.87	8.90	**8.11**	52.33	8.90	11.97
Q	**16.24**	17.20	16.34	27.07	22.29	19.66
E	**15.93**	17.80	16.73	27.11	22.28	21.74
G	**18.03**	19.50	18.53	35.76	24.48	21.23
H	15.87	15.10	**14.26**	33.87	19.37	16.64
I	**8.09**	8.70	8.51	61.34	10.56	12.47
L	**9.79**	9.80	9.80	57.84	12.11	13.40
K	15.77	15.80	**14.64**	22.11	18.31	18.39
M	11.32	**11.30**	11.46	53.58	14.22	14.25
F	10.05	10.20	**10.03**	55.35	11.72	13.12
P	16.69	17.40	**16.10**	29.19	21.51	19.01
S	**16.08**	18.30	16.78	35.19	23.05	19.78
T	15.87	16.00	**15.05**	35.43	21.58	17.86
W	12.17	**11.80**	12.31	52.21	13.43	14.97
Y	**11.51**	13.00	12.06	47.67	14.42	14.07
V	9.89	**9.60**	9.65	58.67	12.43	12.00
win	**10**	3	7	0	0	0
CC	**0.69**	0.68	**0.69**	0.5	0.51	0.59
MAE	**14.11**	14.8	14.89	39.22	19.45	18.07

*The bolds means the best results

### Knowledge retrieval from informative PCPs

The amino acid residues were defined as hydrophobic and hydrophilic using the Kyte-Doolittle index [31]. This definition, which specifies Ala, Cys, Ile, Leu, Met, Phe, and Val as hydrophobic residues, was used in another thermodynamic study on peptides [39]. In the present study, the PCPs that were used to predict RSA of hydrophobic and hydrophilic residues were compared and analyzed.

Lesk *et al*. [40] suggested that surface exposure of hydrophilic residues and burying of hydrophobic residues minimize the free energy of a protein. This concept has been successfully applied in most protein folding studies and protein engineering methods. Nonetheless, there are still some protein folding cases not conforming to this principle. For example, Reidhaar-Olson and Sauer [41] used the λ-repressor to analyze the acceptable substitutions of residues. The results revealed that among most positions on the surface, many positions tolerate substitution of hydrophilic residues with hydrophobic ones and vice versa. Some positions have a strong preference for hydrophilic residues. There are still some results indicating that positions containing exposed hydrophobic residues show a strong preference for hydrophobic residues. Leu12, one of the two exposed hydrophobic residues, in helix 1 of the λ-repressor can be substituted with one of 10 amino acids including six hydrophobic ones. Two of the five exposed positions in α-helix 5 can contain only a hydrophobic residue. At these two positions, Ile84 cannot be substituted and Met87 can be changed only to Leu. This phenomenon indicates that there are some protein folding principles that are unknown to science.

To retrieve more knowledge from our RSA predictors, all the feature sets were compiled according to the binary property: hydrophobic or hydrophilic residues. The RSA predictors were analyzed according to the PCPs used in hydrophobic-residue predictions, hydrophilic-residues predictions, or PCPs used in predictions related to hydrophobic and hydrophilic residues. The results are listed in Additional file 1.

Among the PCPs appearing both in hydrophilic-residue and hydrophobic-residue RSA predictors, BIOV880101 and BIOV880102 were generated from globular proteins. These features—which can be described as “Information value for accessibility; average fraction 35%” and “Information value for accessibility; average fraction 23%”—are the accessibility scales with a different average fraction for different amino acid residues. Because these PCPs are statistical results of RSA, combining these two PCPs within the RSA predictor improved the performance. The other PCPs including the hydrophobicity properties (e.g., GUOD860101, CIDH920103, and MITS02101) and secondary-structure properties (e.g., CHOP780211, MAXF760104, and PALJ810113) suggest that the peptide conformation properties are important for prediction of RSA. Secondary-structure information contributing to prediction of RSA is in agreement with the results of other researchers [11–20], who predicted hydrophilic and hydrophobic residues using single models directly using the secondary structure probability from other secondary structure predictors.

Among the PCPs that appear only in hydrophilic predictors, the PCPs containing 29 parameters were categorized into three types. In the first type, the PCPs correlate with hydrophobicity, such as CORJ870105, JANJ790102, and GEIM800109. This type of PCP constitutes 37% (11/29) of the parameters of all the PCPs appearing only in hydrophilic predictors. The second type is the feature correlating with maintenance of protein structures, such as salt bridge formation and hydrogen-bounding properties. There are three parameters of this type: FAUJ880109, RACS770103, and RACS770103. One study [9] showed that a salt bridge and hydrogen bonding from the side chain are mediated by hydrophilic residues. On the other hand, those residues forming the hydrogen bonds and salt bridges will be buried in the interior of proteins. The PCPs of the third type are the features corresponding to active sites, such as the orientation and electrostatic properties. Those PCPs include FAUJ880102, FAUJ880103, JANJ790102, SIMZ760101, RACS770103, RACS820109, RACS820110, and RADA880103. Although the active-site residues interact with solvent and substrate molecules, these residues are buried. Bartlett *et al*. [8] analyzed 178 active sites of enzymes. In the solvent accessibility analysis, 89% of the catalytic residues showed the RSA less than 30%. Moreover, approximately 50% and 25% of catalytic residues had RSA of 0–10% and 10–20%, respectively; 5% of all catalytic residues had 0% RSA.

According to the PCP analysis of the hydrophilic-residue RSA predictors, except for the first type, which is related to residue hydrophobicity, the other two types are the properties related to characteristics of buried residues. We assumed that the hydrophilic-residue RSA predictors need to use some characteristics of buried residues to estimate the buried status of hydrophilic residues that favor exposure on the protein surface.

Among the 55 PCPs that were used only in hydrophobic-residue RSA predictors, the secondary-structure features constituted 36% (20/55), while the hydrophobicity properties and conformation properties constituted 53% (29/55) and 11% (6/55), respectively. Among the secondary-structure features, the properties related to an α-helix (17 member parameters) represented the majority of the 20 secondary-structure features, while other secondary-structure characteristics (related to β-sheets, γ-turns, and random-coils) represented only 15%. The effects of residue hydrophobicity on protein folding are well studied [42–44], but retrieval of more information from secondary-structure characteristics would be helpful.

According to a study on α-helical structure [45], an α-helix can be exposed on the surface or buried in a protein. Among the α-helix structure features in our study, the position of a residue in the α-helix represents the majority. Those features include URR980103, AURR980113, AURR980117, CHOP780207, MAXF760106, QIAN880108, QIAN880111, RICJ880101, RICJ880111, RICJ880112, and RICJ880115, whereas ROBB760102, ROBB760103, ROBB760104, and ROBB760107 are the characteristics of the α-helix position in a protein. The fact that the residue position characteristics are in the majority indicates that the position of the α-helix is important.

### The hydrophobic spine in α-helices

Because the residue position in an α-helix is important for prediction of the RSA for hydrophobic residues, the influence of a hydrophobic residue position on an α-helix was evaluated. In hydrophilic-residue RSA predictors, we used buried-status characteristics of hydrophilic residues that favor exposure on the surface of proteins. Therefore, we hypothesized that the residue position in an α-helix is related to surface exposure-related characteristics that may also be important for prediction of RSA for hydrophobic residues.

**Fig. 1:**
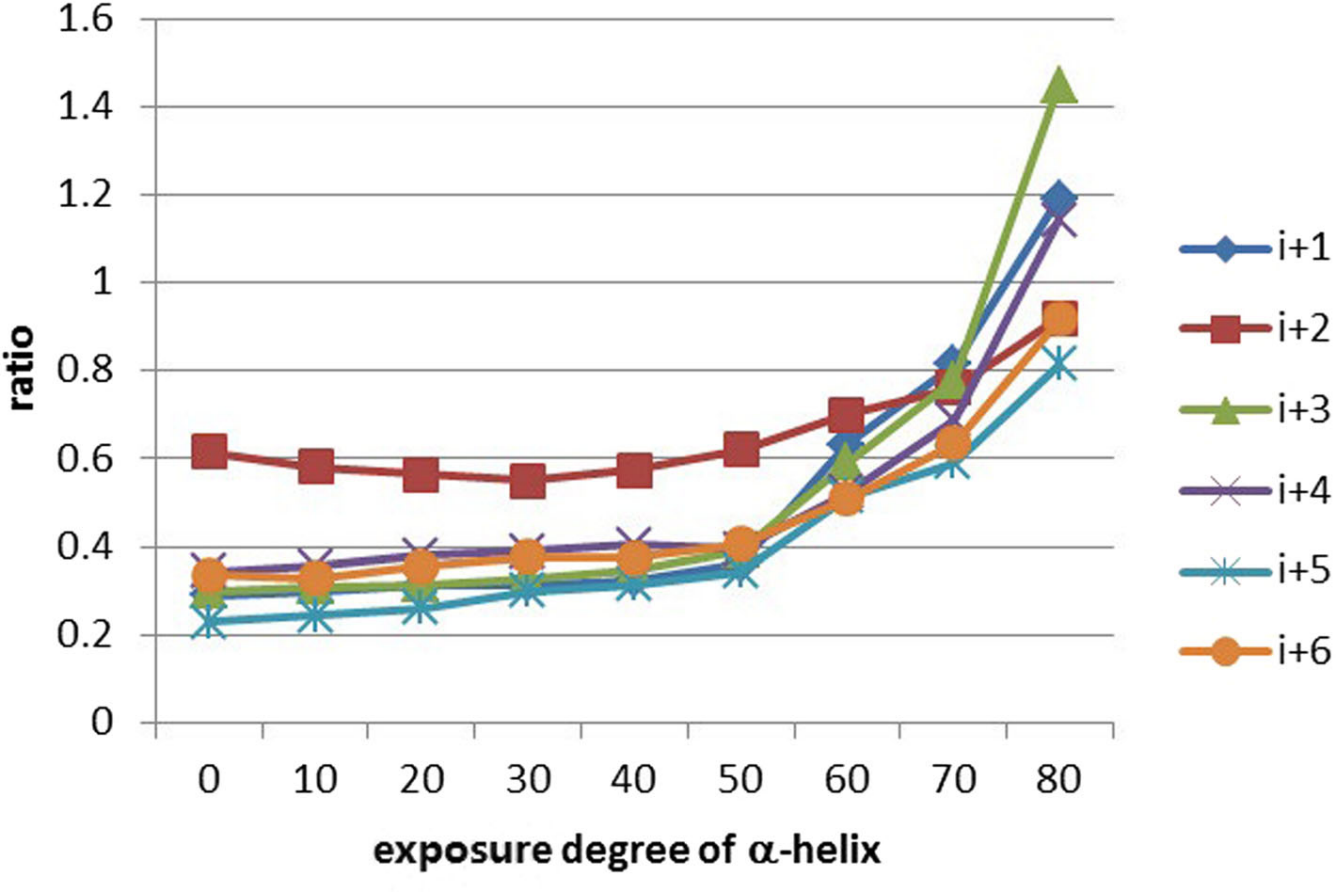
The exposed hydrophobic/hydrophilic neighbor ratios of exposed hydrophobic residues as a function of the exposure degree of an α-helix

**Fig. 2:**
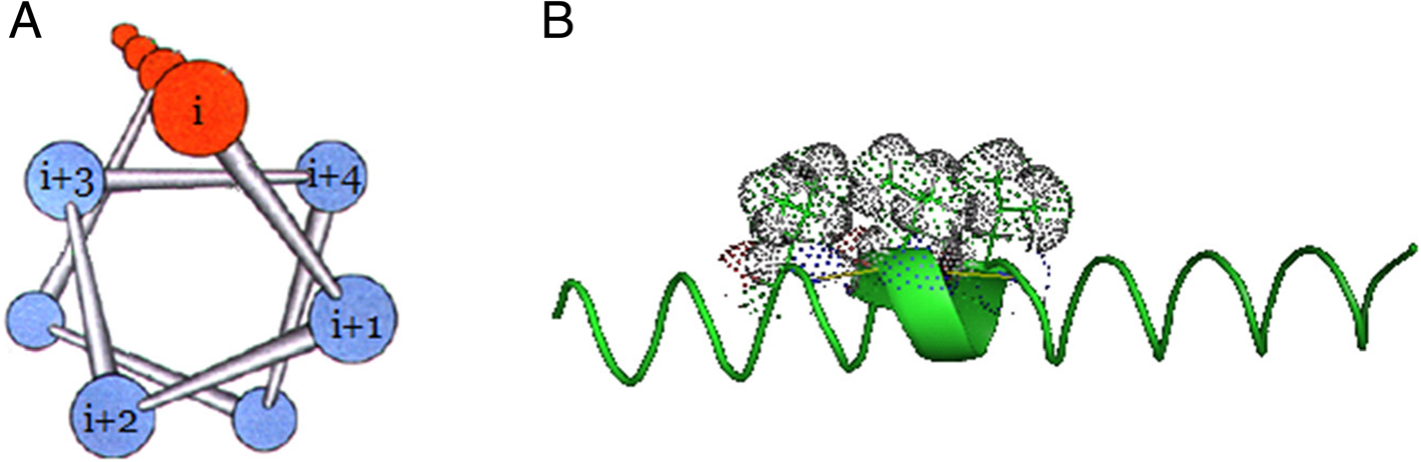
Illustrations of a hydrophobic spine. **a**. The helical wheel of an α-helix. **b**. The hydrophobic spine of an α-helix. The green ribbon means the α-helix. The green sticks are the side chains of the residues constituting the α-helix. Black dots outline the sphere surface of the side chain atoms

To test how the residue position in an α-helix influences the protein, all secondary structures of Barton502 were defined using the DSSP software [24]. All α-helical structures were then sampled, and then we determined their exposure degree. All the residues of α-helices can be classified as exposed hydrophilic residues, buried hydrophilic residues, exposed hydrophobic residues, and buried hydrophobic residues. The exposed hydrophobic residues and their exposed neighbors were predicted by calculations. The exposed hydrophobic/hydrophilic neighbor ratios of exposed hydrophobic residues at various exposure degrees of α-helices are shown in Fig. 1. The neighbors at positions i + 1 to i + 6 were analyzed according to the study by Qian *et al*. [46], who also used two features, QIAN880108 and QIAN880111, in the hydrophobic-residue RSA predictors. The results showed that the ratios remain stable at the α-helix exposure degree less than 50. The i + 2 positions, which are the positions farthest from position i, as shown in Fig. 2a, have the ratio of ~0.6, while the other positions are in the range 0.2–0.4. This finding suggests that an exposed hydrophobic residue favors having exposed hydrophilic residues as neighbors when the α-helix exposure degree is less than 50. This pattern changes when the exposure degree is greater than 60. Positions i + 1, i + 3, and i + 4 show dramatically increasing exposure degree; this finding suggests that the exposed hydrophobic residues prefer to have exposed hydrophobic residues as neighbors when the exposure degree is greater than 60. This result indicates that when exposed hydrophobic residues are located in a highly exposed α-helix, their neighbors are likely to be exposed hydrophobic residues. As shown in Fig. 2b, these hydrophobic residues are arranged on one side of the α-helix. According to the pattern published in reference [47], an α-helix forms a hydrophobic face for contact with interior hydrophobic residues but forms a hydrophilic face for interaction with a solvent. The arrangement of hydrophobic residues observed in the present study is consistent with direct exposure to a solvent. For further evaluation of this effect, we named the set of hydrophobic residues arranged in an α-helix a *hydrophobic spine*.

Hydrophobic spines are different from hydrophobic cores which are packing of hydrophobic residues existing in proteins [48]. Hydrophobic spine taking place on a single a-helix is composed of the adjacent hydrophobic residue contacting. The hydrophobic spine is hypothesized to play two roles. One is to drive the protein-protein or protein-ligand interaction, such as that in the leucine zipper [49]. In the present study, the dataset was collected for analysis of protein folding; therefore, a hydrophobic spine appears to stabilize protein structure.

### Estimating the hydrophobic-spine stability using molecular dynamics simulations

**Fig. 3:**
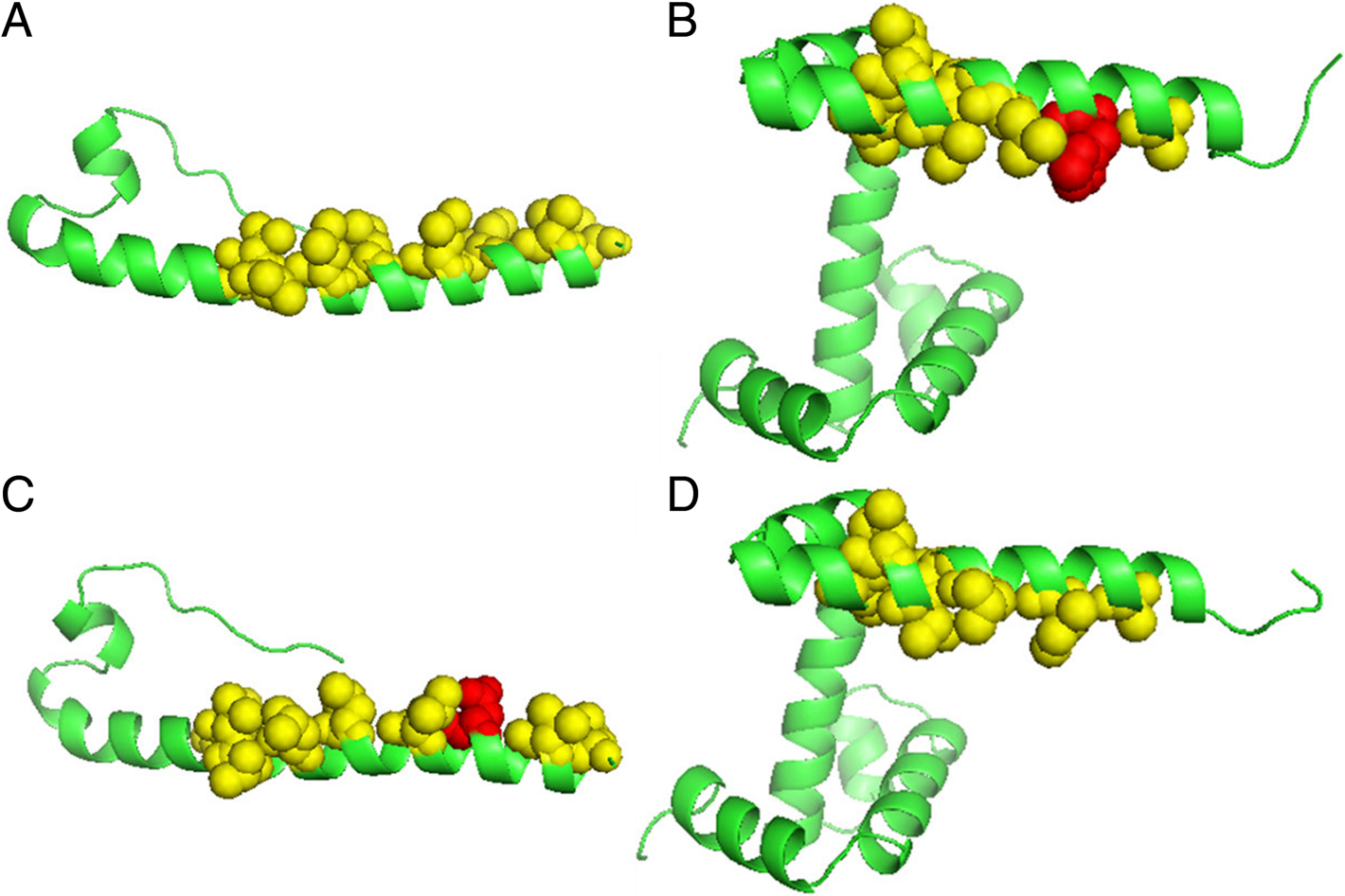
The structures of proteins 1MOF (**a**), 2WRP (**b**), 1MOF_I54D (**c**), and 2WRP_H15I (**d**). The yellow spheres denote the residues constituting the hydrophobic spine. The red spheres are the side chains of hydrophilic residues that interrupt the hydrophobic spine (resulting in an imperfectly hydrophobic spine)

To test the above-mentioned hydrophobic spine hypothesis, whole PDB files of CB513 were scanned, and we chose the proteins that have a perfectly hydrophobic spine and an imperfectly hydrophobic spine. After scanning CB513, 571 α-helices are sampled and 13 hydrophobic spines which contain 4 perfectly and 9 imperfectly hydrophobic spines appear. Among those a-helices having hydrophobic spines, the exposed degrees over than 90% are considered. There are four candidates, 1HUP, 1MOF, 2WRP and 1RPO. The shortest sequences were selected to reduce the simulation computing. 1MOF and 2WRP, which are an extraviral segment of a retrovirus envelope protein and Trp repressor, respectively, were tested here. These two proteins and their mutants are shown using Pymol software (Version:1.8 education) [50]. The secondary structures are shown in “Ribbon” and the residues of hydrophobic spines are emphasis using “Sphere”. The perfect hydrophobic spine appears to extend from L47 to L69 in 1MOF as shown in Fig. 3a. According to the Kyte-Doolittle index, Asp and Glu having the same index, −3.5, were analyzed rather than Lys because the –CH_2_− group is thought to be the hydrophobic interaction contributor [51]. Eisenberg [52] showed that Asp is more hydrophobic than Glu, and Asp was therefore used to mutate I54 which has the highest hydrophobic index in the hydrophobic spine. 2WRP has an imperfectly hydrophobic spine from Ala8 to Leu25. The His residue that is located at position 16 interrupts the continuous hydrophobic spine as shown in Fig. 3b, and an Ile was used to change this His to make this hydrophobic spine perfect. These four proteins were then subjected to molecular dynamics simulations at the temperatures of 300, 400, and 500 K.

**Fig. 4:**
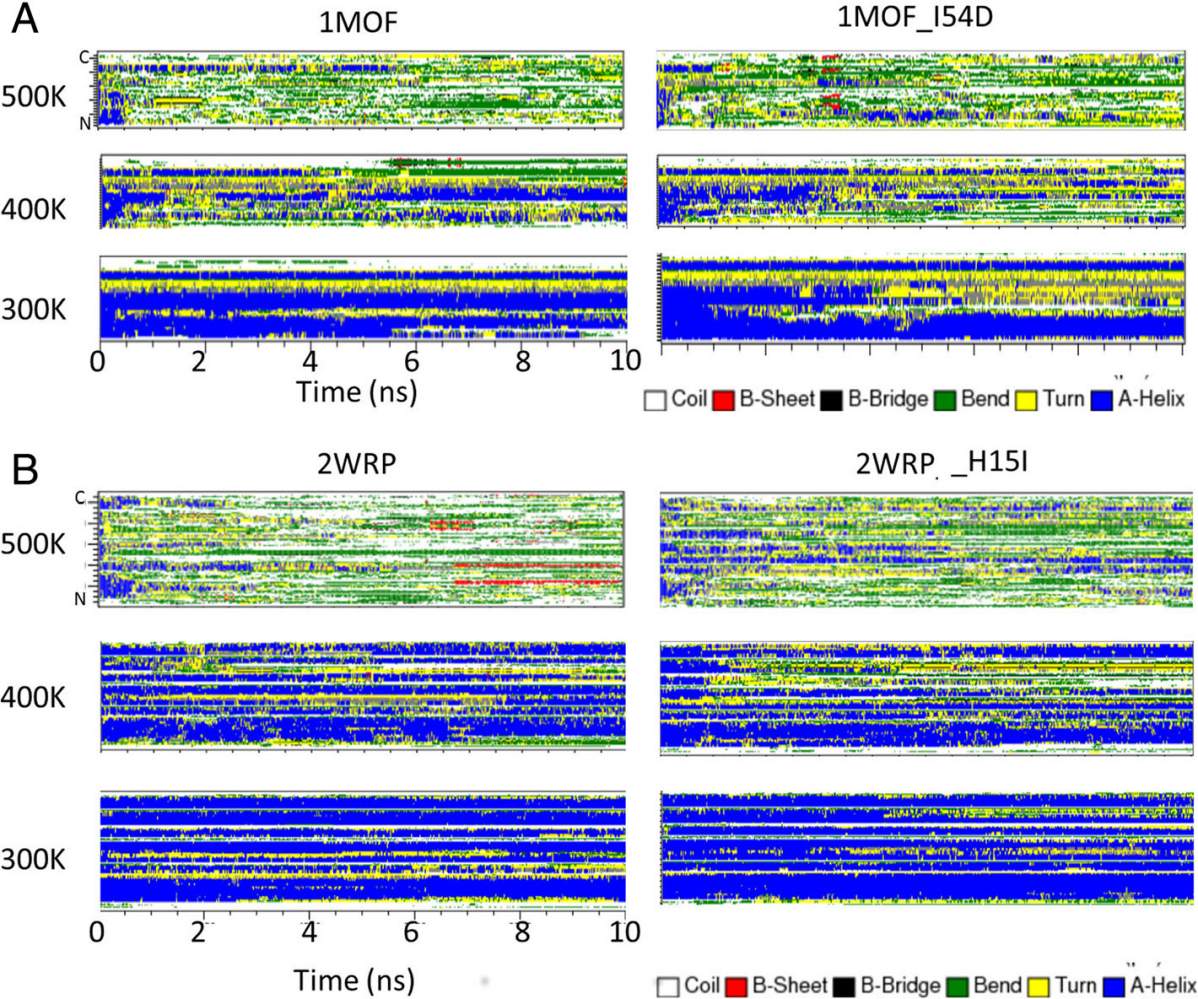
The secondary structure components (shown in different colors) of proteins 1MOF, 1MOF_I54D, 2WRP, and 2WRP_H15I from 10-ns molecular dynamics simulations at the temperatures of 300, 400, and 500 K

Secondary structures were used to estimate the stability of the proteins as shown in Fig. 4. At 300 and 400 K, 1MOF, 1MOF_I54D, 2WRP, and 2WRP_H15I were not in an unfolding state. Sethuraman *et al*. [53] say that an α-helix-rich protein adopts an alternate structure rich in β-sheets during the unfolding process. This β-sheet-rich structure is a molten-globule-like structure. 1MOF, 2WRP, and their mutants have stable α-helical structures at the temperature of 300 K. Although the β-sheet structures appear in the simulations of these four proteins at the temperature of 400 K, those temporary β-sheet structures are not stable, suggesting that these proteins are still at the unfolding initiation stage [53].

In the 500-K simulation of 1MOF, the native structures (which are not refolded structures) during the unfolding process are stable at 0.5 ns, while most of native helical structures of 1MOF_I54D were found to be disrupted at 0.25 ns. Stable β-sheet structures appear at 3 ns of 1MOF_I54D suggesting that this mutant assumes molten-globular structure faster than the wild type does. These results indicate that the protein structure becomes unstable if the hydrophobic spine is disrupted. 2WRP lost its native structures after 0.25 ns, and the molten-globular structures that have stable β-sheets appeared in the molecular dynamics simulation at 6 ns and 500 K. 2WRP_H15I kept the native structure at 1 ns, and there were no emerging β-sheet structures. These results suggest that 2WRP_H15I has a perfectly hydrophobic spine and has more stable structures than 2WRP does.

The average α-helix content analysis for whole protein structures and hydrophobic spines of 1MOF, 1MOF_I54D, 2WRP, and 2WRP_H16I at different temperatures are also carried out by the means of DSSP as shown in Table 5. The other secondary structure information is provided in Additional file 2. Anderson-Darling two sample test applied for determining the difference between the wild-type and mutant protein at the same simulating temperature is calculated using R (version:3.2.5) with kSamples package. The results show the α-helix contents have significant differences except 2WRP and 2WRP_H16I at 500 K. Since the final average α-helix contents of 2WRP and 2WRP_H16I are less than 1.3%, this result is postulated that the α-helices are disrupted at the initial stage of the simulations.

**Table 5:** Average a-helix contents (%) from DSSP analysis for 1mof, 1mof-I54D, 2wrp, and 2wrp-H16I at different temperatures

whole protein						
	300 K	*p*-value	400 K	*p*-value	500 K	*p*-value
1mof	51.27	**>0.001**	22.46	**>0.001**	4.80	**>0.001**
1mof-I54D	43.38		21.81		5.42	
2wrp	65.44	**>0.001**	48.03	**>0.001**	4.62	**>0.001**
2wrp-H16I	61.51		42.41		13.86	
hydrophobic spine regions						
1mof	30.85	**>0.001**	11.61	**>0.001**	3.64	**>0.001**
1mof-I54D	29.95		10.20		1.61	
2wrp	13.71	**>0.001**	11.52	**>0.001**	1.30	0.84
2wrp-H16I	13.26		12.29		1.12	

The boldface indicates the significant difference after Bonferroni correction

We assumed that a hydrophobic spine can prevent a solvent molecule from attacking. A study on human lysozyme [54] revealed that when the hydrophilic residue is exposed on the surface, this residue can interact with the solvent molecules and initiate the two-stage unfolding process. The solvent molecules first disrupt the backbone hydrogen bounds, and then this disruption will attract more attacks by solvent molecules [54, 55]. The hydrophobic-spine characteristics can be applied to protein engineering or may explain why in existing studies the exposed hydrophobic residues make the protein stable. For example, Arc repressor research [56] revealed that Ile84 and Met87 are exposed but cannot be mutated to hydrophilic residues. This may be because these hydrophobic residues are located in the hydrophobic spine.

## Conclusions

RSA and protein folding correlate strongly. Hence, in this study, the aim was to discover knowledge on protein folding using high-performance RSA predictors. Comparing to most existing protein folding characteristic studies which supposed hypotheses and then provided the statistical evidences, this study interpreted the optimal feature sets of the models and discovered *hydrophobic spines* on α-helices which would be helpful to protein engineering enhancing the thermal stability of proteins. Twenty models for different amino acid residues were built here using PSSM, sequence length information, and PCPs which were selected using IBCGA-SVR. The MAE and correlation coefficient of the predictors are 14.11 and 0.69, respectively. Those PCPs were analyzed according to the models. In the hydrophilic-residue models, the buried-status-related characteristics including active-site parameters, hydrogen-bonding characteristics, and salt bridge properties were used. In the hydrophobic-residue models, the secondary structure characteristics are in the majority. After further analysis of these secondary-structure characteristics, the effect of the hydrophobic spine manifests itself. To validate the hydrophobic spine stability, 1MOF and 2WRP, which have a perfectly and imperfectly hydrophobic spine, respectively, were used in molecular dynamics simulations to estimate the structure stability. 1MOF_I54D and 2WRP_H16I that have a disrupted hydrophobic spine and a repaired perfectly hydrophobic spine, respectively, were also simulated for comparison. In the simulations at 300 or 400 K, all these four proteins did not show significant secondary-structure disruption. In the simulations at 500 K, 1MOF and 2WRP_H16I (which have a perfectly hydrophobic spine) were found to have more stable structures than 1MOF_I54D and 2WRP do, which have an imperfectly hydrophobic spine. These results indicate that a hydrophobic spine can help to stabilize protein structure.

## Additional files

Additional file 1: Table S1. The physicochemical properties mined using IBCGA-SVR and used for knowledge discovery. (DOCX 15 kb)
Additional file 2: Table S2. Average secondary structure contents from DSSP analysis for 1mof, 1mof-I54D, 2wrp, and 2wrp-H16I at different temperatures. (XLSX 12 kb)
