# Supplementary material for: A hydrophobic spine stabilizes a surface-exposed α-helix according to analysis of the solvent-accessible surface area
Source: BMC Bioinformatics. 2016 Dec 22;17(Suppl 19):503. doi: 10.1186/s12859-016-1368-z (PMC5259910; doi:10.1186/s12859-016-1368-z)
Supplement: Additional file 1: Table S1. — The physicochemical properties mined using IBCGA-SVR and used for knowledge discovery. (DOCX 15 kb) [file 12859_2016_1368_MOESM1_ESM.docx]

SupTab1. The physicochemical properties mined using IGA-SVR and used for knowledge discovery.

| Both | Only hydrophilic | Only hydrophobic | |
| --- | --- | --- | --- |
| BIOV880101 | CHAM830103 | ARGP820102 | QIAN880108 |
| BIOV880102 | CHOP780206 | ARGP820103 | QIAN880111 |
| BROC820102 | CORJ870105 | AURR980103 | RACS820103 |
| CHAM820102 | DAWD720101 | AURR980113 | RACS820107 |
| CHOP780211 | ENGD860101 | AURR980117 | RICJ880101 |
| CIDH920103 | FASG890101 | BROC820101 | RICJ880111 |
| CORJ870103 | FAUJ880102 | BULH740101 | RICJ880112 |
| GARJ730101 | FAUJ880103 | CHAM830104 | RICJ880115 |
| GUOD860101 | FAUJ880109 | CHAM830108 | ROBB760102 |
| MAXF760104 | FODM020101 | CHOP780207 | ROBB760103 |
| MITS020101 | GEIM800109 | CORJ870106 | ROBB760104 |
| MONM990201 | GUYH850101 | CORJ870107 | ROBB760107 |
| NADH010101 | HOPT810101 | CRAJ730102 | ROBB760109 |
| NADH010103 | JANJ790102 | DESM900101 | ROBB760112 |
| NADH010106 | KUMS000104 | FASG760103 | VHEG790101 |
| NADH010107 | NADH010104 | GEIM800103 | WILM950101 |
| NAKH900106 | NADH010105 | GEIM800110 | WILM950103 |
| NAKH900112 | OOBM850102 | HOPA770101 | WILM950104 |
| NAKH920105 | PARJ860101 | ISOY800106 | WOLS870102 |
| NAKH920108 | QIAN880120 | KOEP990101 | ZIMJ680103 |
| OOBM850104 | RACS770103 | LAWE840101 |  |
| PALJ810113 | RACS820109 | MAXF760105 |  |
| QIAN880115 | RACS820110 | MAXF760106 |  |
| RACS820101 | RADA880103 | MEEJ800101 |  |
| RADA880105 | RICJ880109 | MEEJ810101 |  |
| ROBB760101 | RICJ880110 | MEEJ810102 |  |
| ROBB760106 | SIMZ760101 | NADH010102 |  |
| ROBB760111 | VASM830102 | NAKH900113 |  |
| ROBB790101 | WOLS870103 | NISK800101 |  |
| SUEM840102 |  | NISK860101 |  |
| WERD780104 |  | NOZY710101 |  |
| WILM950102 |  | OOBM850103 |  |
| WOLR810101 |  | PRAM900101 |  |
